# The association of dietary approaches to stop hypertension (DASH) and Mediterranean diet with mental health, sleep quality and chronotype in women with overweight and obesity: a cross-sectional study

**DOI:** 10.1007/s40519-023-01581-0

**Published:** 2023-07-03

**Authors:** Farideh Shiraseb, Atieh Mirzababaei, Elnaz Daneshzad, Darya Khosravinia, Cain C. T. Clark, Khadijeh Mirzaei

**Affiliations:** 1 Department of Community Nutrition, School of Nutritional Sciences and Dietetics, Tehran University of Medical Sciences (TUMS), P.O. Box: 14155-6117, Tehran, Iran; 2 Non-Communicable Diseases Research Center, Alborz University of Medical Sciences, Karaj, Iran; 3 Department of Nutrition, Science and Research Branch, Islamic Azad University, Tehran, Iran; 4 Centre for Intelligent Healthcare, Coventry University, Coventry, CV1 5FB UK

**Keywords:** DASH, Obesity, Mediterranean diet, Circadian rhythm, Mental health, Sleep quality

## Abstract

**Purpose:**

Mental and sleep disorders are global public health problems, especially in Middle Eastern countries, and are significantly associated with circadian rhythm. This study sought to investigate the association between the dietary approaches to stop hypertension (DASH) and Mediterranean diet scores and mental health, sleep quality, and circadian rhythm.

**Methods:**

We enrolled 266 overweight and obese women, and depression, anxiety, and stress scale (DASS) score, Pittsburgh Sleep Quality Index (PSQI), and Morning–Evening Questionnaire (MEQ), were assessed. The Mediterranean and DASH diet score was measured using a validated semi-quantitative Food Frequency Questionnaire (FFQ). The physical activity was evaluated using the International Physical Activity Questionnaire (IPAQ). Analysis of variance and analysis of covariance, chi-square, and multinomial logistic regression tests were used as appropriate.

**Results:**

Our results showed that there was a significant inverse association between adherence to the Mediterranean diet and mild and moderate anxiety scores (*p* < 0.05). In addition, there was an inverse association between adherence to the DASH diet and the risk of severe depression and extremely severe stress scores (*p* < 0.05). Moreover, higher adherence to both dietary scores was associated with good sleep quality (*p* < 0.05). There was a significant relationship between circadian rhythm and the DASH diet (*p* < 0.05).

**Conclusion:**

A significant association exists between a DASH and Mediterranean diet with sleep status, mental health, and chronotype in women of childbearing age with obesity and overweight.

**Level of evidence:**

Level V, Cross-sectional observational study.

## Introduction

Mental disorders, such as anxiety and depression, are global public health problems, especially in Middle Eastern countries [1, 2]. Indeed, according to recent statistics, about 21% of the Iranian adult population is affected [3]. Meanwhile, studies have shown that the prevalence of mental disorders in Iranian women (37.9%) is higher than in men (28.6%) [4]. Stress may lead to adverse consequences on learning ability, while high-stress levels can engender anxiety, behavioural issues, decreased productivity in working time, and job burnout [5–7]. Prolonged stress can lead to depression, which is the leading cause of disability worldwide [8]. Rhythmic patterns of approximately 24 h are circadian rhythms, that are exhibited by most organisms and can optimize and regulate the function of cells, organs, systems, and behavior [9, 10]. Feast–famine and activity–rest within 24-h days affect physiological cycles in a wide kind of processes, including metabolic processes and gastrointestinal function [11]. Sleep is a critical homeostatic mechanism and one of the most robust circadian rhythms that is crucial for functional integrity, modulating morbidity, and regulating circadian hormones [12, 13]. Sleep inadequacy is a public health problem that is associated with weight gain, as well as obesity-related diseases. There are several circadian phase markers, one of which is known as chronotype or circadian preference. Chronotype refers to individual preferences regarding timing of activity and sleep [14]. People can be categorized as morning, intermediate, or evening-type. In contrast with morning, people with evening chronotype may eat breakfast later, skip breakfast, and have a habit of eating late [15–17]. Chronotypes are often associated with food intake, especially among the evening type. Late chronotypes have low adherence to a healthy diet, eat late, have a habit of skipping breakfast, consume fewer fruits and vegetables, and prefer sugary foods/drinks and alcohol. It is well-known that diet quality affects human health status [18–22]. In addition, there is an association between sleep quality and duration and total calorie intake [23, 24], and growing evidence indicates one of the most important risk factors for mental disorder progression is low qualitative diets [25].

The prevalence of obesity in Iranian women was 29.8% in 2016. In addition, there was a significant difference in the prevalence of obesity between Iranian men (15.3%) and women [26]. Since obesity and overweight are related to mental health status, circadian rhythm, and sleep status [27], and on the other hand, Iranian women are more exposed to mental and sleep problems, this is a concern in this population [4, 28]. Many studies show the potential beneficial effects of diet on mental health [29–31]. However, much research on diet and mental health has focussed on single nutrients or food intakes, even though dietary patterns can drastically affect the biological mechanisms of foods, therein confounding their real effects [32]. Dietary patterns are a holistic and comprehensive approach method and are an effective unique method for assessing relationships and understanding the etiologic role between diet and mental health disorders [32, 33]. One of the most healthy dietary patterns in the Mediterranean is characterized by a high intake of plant foods, which has a protective effect against mental health disorders [34, 35]. Another popular healthy dietary pattern is the dietary approaches to stop hypertension (DASH) diet, which is somewhat different from the Mediterranean diet with a higher content of calcium and lower contents of total fat and sodium [35–38]. There is an evident association between hypertension, oxidative stress and inflammation, and impaired mental health, and there is also some evidence that indicates that the mentioned dietary patterns could ameliorate oxidative stress and inflammation [39, 40]. Therefore, we aimed to investigate the impact of the following Mediterranean and DASH-style diets on mental health, circadian rhythm, and sleep quality in people.

## Materials and methods

### Study population

This cross-sectional research was performed on a total of 266 females, with an age range of 18–48 years, who were selected by a multistage cluster random sampling method following referral to the health center in Tehran. Participants were evaluated using the following eligibility criteria and inclusion criteria: healthy overweight (25–29.9 kg/m^2^) and obese (≥ 30 kg/m^2^) women. The exclusion criteria for this study were as follows: conventional medication (including oral contraceptives and medication's effect on sleep or mental status), history of chronic disease, alcohol consumption, pregnancy, lactation, and menopause. In addition, participants were excluded if chronic diseases affected their diet, as well as those who followed any special diet regimen, and those who had any significant changes in their weight in the past year. Participants with daily energy intake below 800 kcal/d or above 4200 kcal/d were also excluded, per standard approaches [41, 42]. Blood samples and anthropometric measurements were taken in the Nutrition and Biochemistry Laboratory of the School of Nutritional and Dietetics at Tehran University of Medical Sciences (TUMS). Moreover, informed written consent was obtained from all participants before the study commencement. The study was approved by the local ethics committee of TUMS (IR.TUMS.VCR.REC. 1399.165).

#### Data collection

Data on demographics, anthropometric measurements, physical activity, Food Frequency Questionnaire (FFQ), depression, anxiety, and stress scale (DASS-21), and Pittsburgh Sleep Quality Index (PSQI) questionnaires were completed by a trained dietitian.

### Dietary intake assessment

Dietary patterns were assessed using a 168-item semi-quantitative FFQ, which was evaluated for reliability and validity in previous studies [43]. The FFQ included a serving-size foods list customarily consumed by Iranians. Participants consumed their usual diet and were asked to report the frequency of consumption of each food item within the prior year on a daily (e.g., bread), weekly (e.g., rice, meat), or monthly basis. Those who reported a total daily energy intake of less than 800 kcal and more than 4200 kcal were excluded from the study (*n* = 2). In addition, those with more than 70 blank FFQ items were excluded from the study (*n* = 0) [44]. Food grams are made by portion sizes converted by using household measures [45]. Dietary intake data were analyzed using the Nutritionist IV software (version 7.0; USA).

### Adherence to DASH-style diet

To assess the DASH-style diet adherence of participants, score assignments were based on conformity foods and nutrients emphasized or minimized in the DASH diet, focusing on eight components: high intake of total grains, vegetables, fruits, dairy, meat, nuts/seeds/legumes, fats/oils, and sweets. In the following, subjects were categorized into deciles of the energy-adjusted intakes of foods and nutrients. Individuals in the lowest decile of total grains, fruits, vegetables, dairy products, nuts, seeds, and legumes were given a score of 1, and those in the highest decile were given a score of 10. It should be noted that the opposite happens when we consider the consumption of sugar-sweetened beverages (SSBs), meat, sweets, sodium, fats, and oils. Therefore, those with the lowest consumption were given a score of 10 and the highest consumption was given a value of 1. A lower intake of sodium, red and processed meats, and sweetened beverages were desired. Finally, the DASH score for each participant was calculated by eight component scores. The highest and lowest DASH scores could be 80 and 8, respectively [46]. Finally, participants were subsequently categorized into quartiles. The first quartile includes scores less equal to 38, the second includes scores between 38 and 44, the third includes scores between 45 and 50, and the fourth includes scores more equal to 50. The number of participants in the first quartile was 67, the second was 67, the third was 66 and the fourth was 66.

### Adherence to the Mediterranean-style diet

Adherence to the Mediterranean diet was determined through scores of studied population compliance with the traditional Mediterranean dietary pattern. For study participants, values of 0 or 1 were assigned to each dietary component by using median cutoffs. Participants whose consumption of vegetables, fruits, legumes, cereals, fish, and the ratio of MUFA to SFA was higher than the median population consumption, were assigned a value of 1, while those whose consumption of these components was below the median were assigned a value of 0. Conversely, consumption of dairy products, red and processed meats was scored a value of 1 if less than the median, or 0 if greater than the median. Hence, the total adherence scores (estimated as the sum of the above-indicated scores of zero and one) varied from 7 to 9 (highest) and 0 (lowest), respectively [47]. Finally, participants were subsequently categorized into tertiles. The first tertile includes scores lower than 4, the second includes scores between 4 and 5, and the third includes scores higher than 5. The number of participants in the first tertile was 88, the second was 89, and the third was 89.

### Mental health

The mental health of participants was determined using a short version questionnaire of the self-report three comorbid DASS-21 (with seven items per subscale) [48]. The validity and reliability of DASS-21 for the Iranian population have previously been confirmed [49]. Each scale has 7 items rated on a 4-point scale. Participants indicate the extent to which they agree with each statement on the 4-point scale, from 0 (did not apply to me at all) to 3 (applied to me very much, or most of the time) over the past week. To calculate scores equivalent to the complete DASS, the score of every scale was multiplied by two. Severe emotional distress was determined by higher scores. For the subgroup of anxiety, mild, moderate, severe, and very severe was considered to be 8–9, 10–14, 15–19, and ≥ 20 scores, respectively. Stress scores ranging from 15 to 18 are considered mild, 19 to 25 moderate, 26 to 33 severe, and ≥ 34 extremely severe. Mild depression is 10–13, moderate depression is 14–20, severe depression is 21–27, and very severe depression is ≥ 28 [49].

### Sleep quality assessment

The PSQI was validated to assess sleep quality. The questionnaire consists of 18 questions on 7 factors, including sleep quality, sleep disorders, sleep duration, delay in falling asleep, SE, sleeping pill use, and disturbances of daily activities due to poor sleep quality. The questionnaire also included five questions answered by people living with the participant, but these are not considered in the scoring. Total scores range from 0 to 21 from the seven components above, with each point being 0–3 points. A score above 5 indicates poor sleep quality [50]. In Iran, the PSQI questionnaire was evaluated for accuracy and validity [51].

### Circadian rhythm assessment

For determining the circadian rhythms of participants who produced peak alertness in the morning or evening, the validated "Morning–Evening Questionnaire" (MEQ) was used. The validity and reliability of MEQ have previously been approved in Iran [52]. The questionnaire consisted of 19 items that focused on sleep patterns and wakefulness habits, with scores ranging from 16 to 86 items. A total score of 70–86 equated to " Definitely morning type ", 59–69 to " Moderately morning type ", 42–58 to Neither type, 31–41 to " Moderately evening type ", and 16–30 to " Definitely evening type " [53]. This questionnaire was collected with a face-to-face interview by a trained interviewer.

### Anthropometric measurements

Anthropometric measures, including body weight, BMI, hip circumference (HC), waist circumferences (WC), waist–hip ratio (WHR), and waist-to-height ratio (WHtR) were measured for all participants. Height was measured, while the participant was standing, without shoes, to the nearest 0.1 cm, by using a non-stretchable tape fixed on a wall, weight was measured with minimal clothing to the nearest 0.1 kg using a Calibrated digital SECA scale (803, German). BMI was calculated by dividing weight in kilograms by the square of height in meters, and WHR and WHtR were calculated by dividing WC by HC and WC by height, respectively.

### Other variables

Using the International Physical Activity Questionnaire (IPAQ), information about light, moderate, and vigorous physical activities were collected. These sub-components were summed by MET scores and weekly MET-minutes (MET-min/week) were calculated; total physical activity (PA) was then reported for all activity categories [54]. Data regarding study population characteristics, such as age, marital status, education, and others, were collected with a questionnaire by a trained dietitian.

### Statistical analysis

With the following formula *n* = (([Z_1-*α*_ *–* *Z*_*1-*β_) ×$\surd$ 1 − *r*^2^]/*r*)^2^ + 2 *α* = 0.05 *β* = 0.95 *r* = 0.20, a sample size of 266 was calculated as being necessary. Data were analyzed by SPSS software version 25 (Chicago, IL, USA). The normality of the dependent variables was confirmed using the Kolmogorov–Smirnov test (*p* > 0.05) and visual inspection of histograms. General characteristics across quartiles of DASH and Mediterranean score tertiles were indicated as mean ± standard deviation (SDs) and mean ± standard errors (SEs), and categorical variables were expressed as numbers and percentages, respectively. A comparison of continuous and categorical variables between the categories of Mediterranean and DASH diet scores was done using analysis of variance (ANOVA) and chi-square tests, respectively. The differences among the categories in each variable were evaluated with an analysis of covariance (ANCOVA), followed by a post hoc test (Bonferroni) when applicable. We used ANCOVA to evaluate the mean difference of quantitative variables among the categories of major dietary patterns and to control the effect of confounders and covariates, such as age, BMI, PA, and energy intake. Multinominal logistic regression models were used to evaluate the association between major dietary patterns and the odds of depression, anxiety, and stress, presented as odds ratio (OR) and 95% confidence interval (CI). In all statistical analyses, statistical significance was accepted at *p* < 0.05, while *p* values of 0.05, 0.06, and 0.07 were considered marginally significant.

## Results

### Study population characteristics

The mean age, weight, height, BMI, and WC of the participants in this cross-sectional study were 36.37 years (SD = 8.43), 80.46 kg (SD = 12.20), 161.18 cm (SD = 5.87), 31.00 kg/m^2^ (SD = 4.37), and 95.19 cm (SD = 16.34), respectively.

The mean DASH and Mediterranean diet scores of participants were 44.32 (SD = 9.17) and 4.50 (SD = 1.60), respectively.

The percentages of mild, moderate, severe, and extremely severe depression, based on the DASS-21 questionnaire, were 9.4%, 12.4%, 7.2%, and 4.5%, respectively, and the percentages of moderate and severe anxiety and stress were 16.8%, 8.4%, 12.1%, and 9.2%, respectively. The percentages of poor sleep quality and good sleep quality, based on the PSQI questionnaire, were 38.4% and 41.1%, respectively. The percentages of definitely evening type, definitely morning type, neither one, moderately evening type, and moderately morning type, based on the MEQ questionnaire, were 0.5%, 2%, 48.2%, 10.6%, and 20.3%, respectively.

### Association between quantitative and categorical variables among categories of DASH and Mediterranean diet

The mean differences between the categories of the Mediterranean and the DASH diet score were analyzed by one-way ANOVA for demographic variables and anthropometric measurements. As shown in Table 1, the women with higher adherence to the Mediterranean diet had higher physical activity (*P* = 0.08), hip circumference (HC) (*P* = 0.09), and family number (*P* = 0.02). In addition, the participants with higher adherence to the DASH diet had higher age (*P* < 0.001) and supplement intake (*P* < 0.001), also there was higher supplement intake (*P* = 0.01) in lower tertiles of the Mediterranean diet score. There was a significant difference in economic status between tertiles before and after adjustment (*P* = 0.03), but some findings were not significant. There were no significant differences in terms of BMI, WC, WHR, education, housing ownership, marital status, and job status (*P* > 0.05) (Table 1).

**Table 1:** Characteristics of the study population among tertiles of Mediterranean diet and quartiles of DASH diet scores in women with overweight and obesity (*n* = 266)

Variables	Tertiles of Mediterranean diet				Quartiles of the DASH diet				
	T1	T2	T3	*P*-value (*P*-value)*	Q1	Q2	Q3	Q4	*P*-value (*P*-value)*
	*N* = 88	*N* = 89	*N* = 89		*N* = 67	*N* = 67	*N* = 66	*N* = 66	
	< 4	4–5	˃ 5		≤ 38	38–44	45–50	≥ 50	
Quantitative variables									
Age (years)	36.31 ± 9.20	37.87 ± 9.33	34.9 ± 7.87	0.10 (0.50)	34.00 ± 8.51^c^	36.11 ± 10.02	37.86 ± 9.55	39.35 ± 7.94^c^	** < 0.001** **(0.01)**
Height (cm)	161.14 ± 5.92	161.21 ± 5.74	160.70 ± 6.10	0.81 (0.54)	161.45 ± 5.90	160.75 ± 5.04	160.94 ± 6.84	161.34 ± 5.65	0.80 (0.20)
Weight (kg)	80.20 ± 11.21	82.70 ± 13.65	80.12 ± 12.30	0.14 (0.20)	82.30 ± 12.12	80.30 ± 14.11	80.81 ± 11.84	80.95 ± 10.81	0.62 (0.34)
PA (MET min/week)	1101.12 ± 2070.84	1070.32 ± 1768.41	1971.14 ± 29.04	0.08(0.16)	1020.720 ± 1715.80	1230.62 ± 2632.81	1147.0 ± 1982.2	1377.77 ± 2106.00	0.76 (0.71)
Family size	3.34 ± 0.01^d^	3.67 ± 0.01	3.50 ± 1.47^d^	**0.02 (0.05)**	3.3 ± 1.05	3.5 ± 1.04	3.55 ± 1.014	3.67 ± 1.34	0.30 (0.41)
BMI (kg/m^2^)	31.07 ± 4.21	31.78 ± 4.46	30.94 ± 4.01	0.20(0.96)	31.75 ± 4.79	31.00 ± 4.79	31.20 ± 4.01	31.0 ± 3.4	0.51 (0.74)
WC (cm)	96.02 ± 15.14	99.7 ± 16.7	95.7 ± 9.2	0.10(0.34)	98.9 ± 10.1	96.8 ± 17.1	95.6 ± 15.9	97.7 ± 18.3	0.62 (0.85)
HC (cm)	112.60 ± 8.42^e^	116.14 ± 10.96^e^	113.42 ± 9.20	0.09(0.22)	114.35 ± 10.91	115.40 ± 12.42	112.64 ± 7.30	114.36 ± 8.57	0.68 (0.61)
WHR	0.80 ± 0.10	0.80 ± 0.10	0.80 ± 0.51	0.41(0.50)	0.82 ± 0.00	0.84 ± 0.00	0.81 ± 0.12	0.84 ± 0.11	0.61 (0.81)
Categorical variables									
Obesity	110 (53.9)^b^	88 (61.5)	23 (54.7)	0.30 (0.21)	47 (40.8)^c^	44 (50)	38 (41.7)	40 (41.6)^c^	0.50 (**0.04**)
Marital status^b^									
Married	60 (29.5)	36 (17.7)	12 (59)	0.67 (0.44)	42 (36.8)	26 (29.5)	22 (24.1)	18 (18.7)	0.21 (0.62)
Single	143 (70.4)	107 (52.7)	30 (14.7)		72 (63.1)	62 (29.5)	69 (75.8)	78 (81.2)	
Education				0.11 (0.50)					0.51 (0.10)
Illiterate	2 (0.9)	0 (0)	2 (0.9)		0 (0)	2 (2.2)	2 (2.1)	0 (0)	
Undergraduate	27 (13.3)	17 (8.3)	5 (2.46)		13 (11.4)	9 (10.2)	13 (14.2)	14 (14.5)	
Diploma	59 (29.0)	51 (25.1)	13 (6.4)		35 (30.7)	33 (37.5)	26 (28.5)	29 (30.2)	
Master and upper	115 (56.6)	75 (36.9)	22 (10.8)		66 (57.8)	44 (50)	50 (54.9)	53 (55.2)	
Housing ownership	115 (56.9)	96 (67.6)	22 (55%)	0.14 (0.35)	74 (64.3)	44 (51.1)	52 (57.7)	63 (67.0)	0.16 (0.11)
Supplement intake	77 (42.7)^d^	55 (47.4)	26 (70.2)^d^	**0.01 (0.02)**	32 (31.3)^c^	33 (45.8)	44 (55)	49 (61.25)^c^	** < 0.001** **(0.04)**
Smoking	14 (6.7)	9 (6.2)	4 (9.7)	0.74 (0.90)	16 (13.9)^c^	4 (4.5)	3 (3.2)	4 (4.2)^c^	**0.006 (0.01)**
Economic status				**0.01(0.03)**					**0.05 (0.01)**
Poor level	40 (45.5)	19 (21.6)	29 (33.0)		17 (19.3)	19 (21.6)	28 (31.8)	24 (27.3)	
Moderate level	110 (60.4)	34 (18.7)	38 (20.9)		57 (31.3)	48 (26.4)	36 (19.8)	41 (22.5)	
Good level	36 (41.4)	19 (21.8)	32 (36.8)		105 (35.2)	80 (14.8)	84 (22.7)	89 (27.3)	
Job-status				0.82 (0.61)					0.39(0.41)
Employed	85 (53.8)	32 (20.3)	41 (25.9)		51 (32.3)	34 (21.5)	31 (19.6)	42 (26.6)	

Bold numbers are the *p* values < 0.05

WHR: Waist to Hip Ratio, BMI: body mass index; DASH: Dietary approaches to stop hypertension; SD: standard deviation, T: tertile; Q: quartile

^a^Mean ± SD, ^b^ n (%), ^c^ association between quartiles 1 and 4 of DASH diet adherence, ^d^ association between tertile 1 and 3 of Mediterranean diet adherence, ^e^ association between tertile 1 and 2 of Mediterranean diet adherence

The Chi-square or Fisher’s exact tests were used for comparison of categorical variables and the one-way analysis of variance (ANOVA) and analysis of covariance (ANCOVA) was used for the comparison of continuous variables

*Adjusted for age, BMI, and physical activity. BMI considers as collinear variables that didn’t adjust for anthropometric measurement

Obesity, BMI > 29.9

The individuals with higher adherence to the DASH diet had lower smoking incidence (*P* = 0.006), as compared to the low DASH diet group. After adjustment for confounders, including age, BMI, and physical activity, a significant difference in obesity (*P* = 0.04), smoking (*P* = 0.01), supplement intake (*P* = 0.04), and economic status (*P* = 0.01) was observed (Table 1).

### Dietary intakes

The dietary intakes of the participants across dietary DASH score quartiles and tertiles of the Mediterranean diet are shown in Table 2.

**Table 2:** Dietary intake among tertiles (T) of the Mediterranean diet and quartiles (Q) categories of the DASH diet in women with overweight and obesity (*n* = 266)

Variables	Tertiles of the Mediterranean diet				Quartiles of the DASH diet				
	T1	T2	T3	*P*-value*	Q1	Q2	Q3	Q4	*P*-value*
	*N* = 88	*N* = 88	*N* = 89		*N* = 67	*N* = 67	*N* = 66	*N* = 66	
	< 4	4–5	> 5		≤ 38	38–44	45–50	≥ 50	
	Mean ± SE				Mean ± SE				
Energy and macronutrients									
Energy (kcal)	2480.9 ± 55.31	2793.9 ± 66.40	2846.9 ± 122.62	**< 0.001**	2648.0 ± 75.01	2565.5 ± 86.11	2540.9 ± 84.74	2765.0 ± 82.40	**0.03**
Fat energy (%)	31.80 ± 0.50	32.51 ± 0.52	37.34 ± 1.70	**0.01**	34.54 ± 2.00	38.62 ± 0.91	32.22 ± 0.84	30.04 ± 0.64	**< 0.001**
Protein-energy (%)	13.51 ± 0.20	14.40 ± 0.24	13.81 ± 0.71	**0.01**	14.2 ± 10.34	12.96 ± 0.82	13.87 ± 0.32	14.69 ± 0.22	0.54
Carbohydrate energy (%)	57.43 ± 0.53	56.16 ± 0.50	51.90 ± 1.84	**0.01**	53.62 ± 0.94	50.87 ± 2.24	57.09 ± 0.97	59.06 ± 0.74	**< 0.001**
Micronutrients									
Zinc (mg/d)	12.85 ± 0.12	14.04 ± 0.21	13.7 ± 20.41	**0.01**	12.91 ± 5.10	12.68 ± 4.45	12.97 ± 5.15	15.06 ± 4.38	**< 0.001**
Calcium (mg/d)	1198.1 ± 27.81	1341.9 ± 33.21	1384.0 ± 61.80	**0.001**	1240.7 ± 37.11	1205.8 ± 42.21	1216.8 ± 41.41	1417.1 ± 40.80	**< 0.001**
Mg (mg/d)	451.80 ± 6.81	496.90 ± 8.21	5247.01 ± 15.30	**< 0.001**	434.80 ± 8.61	452.50 ± 9.91	484.81 ± 9.70	537.04 ± 9.54	**< 0.001**
Iron (mg/d)	26.13 ± 1.30	26.91 ± 1.60	26.62 ± 12.90	0.94	30.4 ± 51.77	25.90 ± 2.04	23.20 ± 1.91	25.31 ± 1.90	**0.05**
Copper (mg/d)	1.92 ± 0.03	2.0 ± 0.03	2.2 ± 0.06	**< 0.001**	1.92 ± 0.83	1.91 ± 0.65	1.93 ± 0.77	2.31 ± 0.62	**< 0.001**
Selenium (mcg/d)	130.72 ± 2.21	124.65 ± 2.70	113.02 ± 5.01	**< 0.001**	125.24 ± 49.66	123.08 ± 47.50	124.75 ± 55.10	133.02 ± 46.13	0.84
Vitamin A (RE/d)	651.20 ± 23.81	862.65 ± 28.40	987.54 ± 52.91	**< 0.001**	644.32 ± 4 ± 31.61	678.30 ± 35.90	775.81 ± 35.24	978.41 ± 34.78	**< 0.001**
Vitamin E (mg/d)	17.24 ± 0.57	16.31 ± 0.66	18.32 ± 1.24	0.30	15.2 ± 0.7	18.7 ± 0.8	17.4 ± 0.8	17.2 ± 0.8	**0.02**
Beta carotene (mg/d)	4082.8 ± 208.5	55,905.1 ± 249.1	7771.1 ± 463.6	**< 0.001**	3993.0 ± 277.7	4345.96 ± 315.74	5213.3 ± 309.1	7161.78 ± 304.84	**< 0.001**
Vitamin C (mg/d)	165.61 ± 6.90	206.22 ± 8.31	242.65 ± 15.50	**< 0.001**	155.8 ± 9.21	183.97 ± 10.40	183.84 ± 10.21	237.85 ± 10.11	**< 0.001**
Thiamin (mg/d)	2.11 ± 0.04	2.14 ± 0.03	1.96 ± 0.05	**0.003**	2.13 ± 0.03	2.17 ± 0.04	2.12 ± 0.04	2.14 ± 0.0	0.85
Niacin (mg/d)	26.33 ± 0.41	26.44 ± 0.50	26.65 ± 0.90	0.91	27.16 ± 0.50	25.55 ± 0.60	25.65 ± 0.61	26.65 ± 0.60	0.21
Riboflavin (mg/d)	2.18 ± 0.04	2.36 ± 0.04	2.47 ± 0.09	**0.005**	2.21 ± 0.05	2.13 ± 0.06	2.24 ± 0.05	2.52 ± 0.05	**< 0.001**
Pyridoxine (mg/d)	2.00 ± 0.03	2.31 ± 0.03	2.41 ± 0.06	**< 0.001**	2.06 ± 0.04	2.14 ± 0.04	2.14 ± 0.04	2.47 ± 0.04	**< 0.001**
Cobalamin (mcg/d)	4.01 ± 0.15	4.82 ± 0.15	41.08 ± 0.30	**0.001**	4.35 ± 0.2	4.10 ± 0.23	4.24 ± 0.2	4.65 ± 0.2	0.34
Folate (mcg/d)	608.8 ± 7.74	629.8 ± 9.24	652.6 ± 17.11	**0.03**	601.8 ± 10.13	612.6 ± 11.50	603.0 ± 11.21	668.2 ± 11.21	**< 0.001**
Biotin (mg/d)	34.7 ± 0.90	40.8 ± 1.12	47.0 ± 2.01	**< 0.001**	32.60 ± 1.20	34.49 ± 1.311	40.35 ± 1.3	46.85 ± 1.31	**< 0.001**
Dietary fiber (g/d)	46.2 ± 1.11	47.3 ± 1.31	53.6 ± 2.61	**0.03**	46.02 ± 26.13	45.73 ± 19.684	44.61 ± 19.19	53.14 ± 17.37	**< 0.001**
SFA (g/d)	28.8 ± 0.54	28.4 ± 0.64	25.8 ± 1.11	0.06	29.58 ± 0.70	29.18 ± 0.70	28.15 ± 0.70	26.75 ± 0.70	**0.04**
MUFA (g/d)	32.4 ± 0.60	31.5 ± 0.71	31.7 ± 1.11	0.61	32.7 ± 0.88	33.3 ± 0.95	32.4 ± 0.95	29.8 ± 0.81	0.03
PUFA (g/d)	20.6 ± 0.52	18.8 ± 0.69	21.7 ± 1.13	**0.03**	19.86 ± 0.71	21.73 ± 0.80	20.25 ± 0.78	18.80 ± 0.78	0.07
Linoleic (mg/d)	18.0 ± 0.54	16.1 ± 0.67	18.5 ± 1.12	**0.03**	17.18 ± 0.69	19.06 ± 0.72	17.63 ± 0.72	16.04 ± 0.72	**0.03**
Linolenic (mg/d)	1.18 ± 0.04	1.19 ± 0.04	1.41 ± 0.08	**0.05**	1.22 ± 0.05	1.28 ± 0.06	1.19 ± 0.06	1.16 ± 0.5	0. 50
EPA (mg/d)	0.02 ± 0.00	0.039 ± 0.00	0.043 ± 0.00	**< 0.001**	0.24 ± 0.00	0.28 ± 0.01	0.03 ± 0.00	0.03 ± 0.00	**0.04**
DHA (mg/d)	0.07 ± 0.00	0.12 ± 0.00	0.14 ± 0.01	**< 0.001**	0.83 ± 0.01	0.09 ± 0.01	0.09 ± 0.10	0.12 ± 0.10	**0.04**
Caffeine (mg/d)	156.32 ± 10.33	159.34 ± 12.40	118.22 ± 23.01	0.20	171.7 ± 209.91	15.5 ± 136.34	138.9 ± 96.03	146.7 ± 106.64	0.41

Bold numbers are the *p* values < 0.05

RE: Retinol equivalent, EPA: Eicosapentaenoic acid, DHA: Docosahexaenoic acid; MUFA: monounsaturated fatty acid; PUFA: polyunsaturated fatty acid; SFA: saturated fatty acid

*Component of dietary pattern adjusted for BMI, age, and PA by ANCOVA test

*Macronutrients and micronutrients adjusted for energy intake by ANCOVA test

Fat intake was significantly higher in the lowest DASH quartiles compared to the highest quartiles (98.34 vs 88.82 g/day, *P* < 0.05). In addition, the women with higher adherence to the tertile Mediterranean diet had higher intake of protein (*P* < 0.001), minerals (*P* = 0.001), vitamins (*P* < 0.05), and dietary fibers (*P* = 0.03).

### Association between DASS total score, subscales, PSQI, and MEQ scores among tertiles of the Mediterranean and DASH diet

The differences between the tertiles and quartiles' adherence to the Mediterranean and the DASH diet were analyzed by ANCOVA in different models for DASS total score, depression, anxiety, stress, MEQ, and PSQI.

As shown in Table 3, there were no significant differences in tertiles of the Mediterranean diet and DASS total score, depression, anxiety, stress, and MEQ in the crude and adjusted models (*P* > 0.05) (Table 3). In the crude model, the women with higher adherence to the Mediterranean diet had a lower PSQI score (*P* = 0.05), but this association was attenuated after adjustment.

**Table 3:** DASS total score and subscales of study population among tertiles of Mediterranean diet and quartiles (Q) categories of DASH diet scores (*n* = 266)

Variables	Model	Tertiles of the Mediterranean diet				Quartiles of the DASH diet				
		T1	T2	T3	*P-value	Q1	Q2	Q3	Q4	*P*-value*
		*N* = 89	*N* = 88	*N* = 88		*N* = 66	*N* = 66	*N* = 67	*N* = 67	
		< 4	4–5	> 5		≤ 38	38–44	45–50	≥ 50	
						mean ± SD				
DASS-21 total Score	Crude	38.60 ± 23.87	33.96 ± 23.77	38.38 ± 25.10	0.31	40.93 ± 25.29	32.95 ± 22.05	36.91 ± 25.11	36.32 ± 23.26	0.35
	1	38.75 ± 2.06	33.65 ± 2.42	38.61 ± 4.03	0.22	41.37 ± 3.42	33.17 ± 3.87	36.99 ± 2.80	35.72 ± 2.71	0.24
	2	38.42 ± 2.40	30.88 ± 2.4	39.91 ± 4.63	0.15	45.91 ± 3.61^	30.41 ± 3.84^	35.44 ± 3.41	33.02 ± 3.14	**0.01**
	3	38.50 ± 2.51	30.94 ± 3.1	40.77 ± 4.70	0.10	45.88 ± 3.60^	30.76 ± 3.81^	35.73 ± 3.41	32.55 ± 3.21	**0.01**
	4	38.53 ± 2.54	29.89 ± 4.1	36.33 ± 3.31	0.10	46.27 ± 3.6^	30.41 ± 3.80^	35.71 ± 3.41	33.27 ± 3.20	**0.01**
Depression subscale score	Crude	11.35 ± 9.35	9.21 ± 9.09	11.55 ± 10.07	0.15	12.66 ± 10.10	9.01 ± 8.06	10.28 ± 9.85	10.27 ± 9.19	0.12
	1	11.34 ± 0.80	9.07 ± 0.94	11.80 ± 1.56	0.14	12.92 ± 1.16	9.07 ± 1.18	10.27 ± 1.11	9.95 ± 1.06	0.12
	2	11.30 ± 0.90	8.59 ± 1.2	12.62 ± 1.85	0.10	14.64 ± 1.42^	8.28 ± 1.55^	974 ± 1.30	9.73 ± 1.24	**0.01**
	3	11.34 ± 1.03	8.61 ± 1.2	12.70 ± 1.91	0.10	14.64 ± 1.41^	8.43 ± 1.55^	986 ± 1.30	9.52 ± 1.35	**0.01**
	4	11.33 ± 1.04	7.86 ± 1.7	11.08 ± 1.34	0.24	14.74 ± 1.50^	8.35 ± 1.54^	9.86 ± 1.34	9.69 ± 1.35	**0.01**
Anxiety subscale score	Crude	10.57 ± 8.15	9.7 ± 8.09	11.11 ± 7.81	0.54	10.39 ± 8.50	9.55 ± 7.22	10.82 ± 8.54	10.42 ± 7.91	0.80
	1	10.63 ± 0.69	9.69 ± 0.81	11.13 ± 1.36	0.51	10.47 ± 1.06	9.72 ± 1.04	10.86 ± 0.90	10.29 ± 0.90	0.80
	2	10.14 ± 0.86	8.59 ± 1.2	12.62 ± 1.84	0.33	11.32 ± 1.24	8.75 ± 1.20	10.63 ± 1.1	8.69 ± 1.06	0.22
	3	10.13 ± 0.81	8.66 ± 1.0	11.49 ± 1.51	0.24	11.29 ± 1.21	8.86 ± 1.20	10.79 ± 1.15	8.5 ± 1.04	0.21
	4	10.13 ± 0.80	7.99 ± 1.3	10.43 ± 1.02	0.30	11.31 ± 1.20	8.64 ± 1.20	8.64 ± 1.20	10.77 ± 1.11	0.21
Stress subscale score	Crude	16.67 ± 10.12	15.03 ± 9.56	15.72 ± 11.35	0.45	17.87 ± 10.41	14.38 ± 9.72	15.80 ± 10.4	15.62 ± 9.51	0.20
	1	16.77 ± 0.87	14.89 ± 1.02	15.67 ± 1.70	0.32	17.98 ± 1.26	14.37 ± 1.28	15.85 ± 1.21	15.47 ± 1.15	0.21
	2	16.96 ± 1.08	13.66 ± 1.22	16.15 ± 19.01	0.11	19.95 ± 1.51^	15.06 ± 1.41^	13.36 ± 1.60	14.58 ± 1.3	**0.01**
	3	17.03 ± 1.07	13.66 ± 1.31	16.57 ± 2.01	0.12	19.94 ± 1.50^	15.07 ± 1.42^	13.46 ± 1.60	14.51 ± 1.37	**0.01**
	4	17.06 ± 1.01	14.03 ± 1.70	14.8 ± 1.42	0.20	20.20 ± 1.50^	13.41 ± 1.62^	15.08 ± 1.4	14.70 ± 1.3	**0.01**
PSQI score	Crude	6.57 ± 3.85	5.62 ± 3.26	5.59 ± 2.42	0.05	6.43 ± 3.65	6.42 ± 3.94	6.23 ± 3.09	5.36 ± 3.42	0.17
	5	5.55 ± 0.51	4.71 ± 0.59	5.16 ± 1.04	0.50	6.25 ± 0.51^	6.14 ± 0.59	6.09 ± 0.65	5.01 ± 0.48^	0.07
MEQ score	Crude	51.88 ± 10.40	53.02 ± 9.25	53.68 ± 9.22	0.40	49.96 ± 8.89^	10.92 ± 1.31	9.66 ± 1.07^	55.84 ± 9.38	**< 0.001**
	**6**	52.33 ± 0.94	53.69 ± 1.11	53.41 ± 1.69	0.61	50.88 ± 0.99^	50.61 ± 1.2	53.55 ± 1.06	55.32 ± 1.11^	**0.007**

Bold numbers are the *p* values < 0.05

DASS: Depression, anxiety, stress questionnaire, MEQ: Morningness–Eveningness questionnaire, PSQI: Pittsburgh Sleep Quality Index, T: tertile, Q: quartile

Model 1 was adjusted for age and energy intake

Model 2 was further adjusted for marital status, educational level, family size, housing ownership status, history of smoking, job status, physical activity, and supplement consumption

Model 3 was further adjusted for dietary intake of omega-3 fatty acids

Model 4 was further adjusted for body mass index

Model 5 was adjusted for age and physical activity and BMI, energy intake, marriage status, educational level, family size, housing ownership, economic status

Model 6 was adjusted for age and physical activity and BMI* P value less than 0.05 was considered statistically significant, 0.05–0.07 consider marginally significant

The *P*-value for the trend across increasing tertiles of a Mediterranean diet and quartile categories of DASH diet scores was calculated using a multivariate analysis of variance by considering the categories as ordinal variables

^^^Association between tertiles or quartiles by the Bonferroni post hoc test

There were no significant differences in quartiles of the DASH diet and anxiety in the crude model and model 4 (adjusted for age and energy intake, marital status, educational level, family size, ownership status, history of smoking, incoming status, physical activity, supplement use, dietary intake of omega-3 fatty acids, and BMI) (*P* > 0.05).

The women with higher adherence to the DASH diet had significantly lower DASS total scores, depression, and stress scores (*P* < 0.05). In Table 3, participants with higher adherence to the DASH diet had a marginally significantly decreased PSQI in model 5 after adjustment for age and physical activity, BMI, energy intake, marriage status, educational level, family size, housing ownership, and economic status, (*P* = 0.07), and a significantly decreased MEQ score in model 6 (*P* < 0.05), which was adjusted for age, physical activity, and BMI.

### Relationship of DASS-21 subscales, sleep quality, and chronotype status with adherence to DASH and the Mediterranean diet

The associations between adherence to a DASH diet and a Mediterranean diet with DASS-21 depression, anxiety, stress subscale, sleep quality, and chronotype status are shown in Table 4.

**Table 4:** Association of mental health subscales, sleep quality status, and chronotypes with DASH and Mediterranean diet (*n* = 266)

Variable	Mediterranean diet						DASH diet					
	Crude model			Adjust model			Crude model			Adjust model		
	OR	0.95% CI	*P*-value	OR	0.95% CI	*P*-value	OR	0.95% CI	*P*-value	OR	0.95% CI	*P*-value
Stress subscale												
Mild	0.85	0.68,1.06	0.30	0.90	0.70,1.16	0.41	0.98	0.94,1.01	0.30	0.98	0.93,1.02	0.32
Moderate	0.89	0.72,1.09	0.20	0.91	0.72,1.16	0.42	1.00	0.96,1.04	0.82	0.97	0.93,1.01	0.22
Severe	0.94	0.75,1.19	0.65	0.91	0.69,1.19	0.45	0.99	0.95,1.03	0.73	0.97	0.92,1.02	0.21
Extremely severe	1.07	0.79,1.46	**0.003**	0.95	0.67,1.36	0.70	0.96	0.90,1.01	0.12	0.92	0.85,0.99	**0.02**
Depression Subscale												
Mild	0.87	0.69,1.08	0.21	0.85	0.66,1.10	0.25	0.98	0.94,1.02	0.44	0.96	0.91,1.00	0.09
Moderate	0.92	075,1.12	0.40	0.93	0.73,1.18	0.53	0.98	0.94,1.01	0.36	0.97	0.93,1.01	0.20
Severe	0.92	0.71,1.19	**0.03**	0.91	0.69,1.21	0.54	0.97	0.92,1.01	0.22	0.94	0.89,0.99	**0.04**
Extremely severe	0.95	0.70,1.28	0.72	0.89	0.63,1.26	0.57	0.98	0.93,1.03	0.42	0.96	0.90,1.02	0.25
Anxiety Subscale												
Mild	0.82	0.63,1.08	0.14	0.82	0.59–1.14	0.20	0.97	0.93,1.02	0.34	0.97	0.91,1.02	0.33
Moderate	0.82	0.67,0.99	**0.04**	0.77	0.61–0.97	**0.03**	0.99	0.96,1.02	0.65	0.97	0.94,1.01	0.32
Severe	1.27	0.99,1.62	**0.04**	0.92	0.91–0.99	**0.03**	1.01	0.97,1.05	0.51	0.99	0.95,1.04	0.90
Extremely Severe	0.92	0.73,1.15	0.43	0.93	0.71–1.22	0.64	0.90	0.95,1.03	0.80	0.97	0.93,1.02	0.31
Sleep Quality												
Good sleep quality	1.05	1.00,1.08	**0.02**	1.01	1.01,1.06	**0.04**	1.16	1.05,1.29	**0.04**	1.02	1.03,1.14	**0.01**
Chronotype												
Completely evening type	0.65	0.41,1.04	0.07	0.66	0.26,1.66	0.35	1.06	0.91,1.24	0.30	1.11	0.93,1.32	0.20
Completely morning type	0.65	0.41,1. 04	0.07	0.69	0.41,1.13	0.11	1.09	1.01,1.18	**0.03**	1.10	1.0,1.19	**0.04**
Relatively evening type	0.82	0.66,1.01	0.07	0.83	0.67,1.03	0.14	1.09	0.92,0.99	**0.04**	0.95	0.92,0.99	**0.02**
Relatively morning type	1.09	0.93,1.28	0.20	1.10	0.93,1.29	0.22	1.02	0.99,1.05	0.09	1.01	0.98,1.04	0.30

Bold numbers are the *p* values < 0.05

*Adjusted for age, PA, BMI, energy intake, smoking, supplement intake, economic status

OR 0.95% CI *P*-value

^a^Normal group between the subgroup of DASS and chronotype consider the references group. Poor sleep quality considers a reference group in sleep quality

^b^The odds ratio (OR) has been reported

Pattern scores were tested by multinominal logistic regression

*P* < 0.05 consider significant, and 0.05−0.07 consider marginally significant

The results of multinomial logistic regression analysis in the crude model showed that people with higher adherence to the Mediterranean diet had lower odds of severe depression (OR = 0.92, 95% CI 0.71,1.19, *P* = 0.03), as compared with the normal group; this became non-significant once adjusted for confounders (*P* = 0.5). However, no relationship was observed between the Mediterranean diet and mild, moderate, and severe depression after adjusting for age, PA, and BMI (*P* > 0.05). There was a significant association with adherence to the Mediterranean diet in the crude model, that remained after adjustment, between moderate anxiety (OR = 0.77, 95% CI 0.61,0.97, *P* = 0.03) and severe anxiety (OR = 0.92, 95% CI 0.91,0.99, *P* = 0.03). Adherence to the Mediterranean diet was associated with increased odds of good sleep quality compared with poor sleep quality (OR = 1.05, 95% CI 1.00,1.08, *P* = 0.02), even after controlling for potential confounders (OR = 1.01, 95% CI 1.01,1.06, *P* = 0.04). Although we found a marginal association between adherence to the Mediterranean diet and reduce odds of relatively evening type compared with the normal group (OR = 0.82, 95% CI 0.66,1.01, *P* = 0.07), after adjustment, no significant association was evident (*P* = 0.1), Table 4**.**

Adherence to the DASH diet had increased odds of good sleep quality compared with poor sleep quality in the crude model (OR = 1.16, 95% CI 1.05,1.29, *P* = 0.04), even after controlling for potential confounders (OR = 1.02, 95% CI 1.03,1.14, *P* = 0.01). There was a significant association between adherence to the DASH diet and reduced odds of relatively evening type, compared with the normal group (OR = 0.95, 95% CI 0.92,0.99, *P* = 0.02). After controlling for confounders, an increased odds of completely morning type compared with the normal group (OR = 1.10, 95% CI 1.0,1.19, *P* = 0.04) was seen (Table 4). In other words, there was no statistically significant correlation between DASH score and reduced anxiety in individuals, mild anxiety (OR = 0.97, 95% CI 0.93,1.02, *P* = 0.34), moderate anxiety (OR = 0.97, 95% CI 0.94,1.01, *P* = 0.32), or severe anxiety (OR = 0.99, 95% CI 0.95,1.04, *P* = 0.90) after adjustment. An inverse association was also seen between adherence to the DASH diet and severe depression. Individuals with greater adherence to the DASH diet had 6% lower odds of severe depression, compared with the normal group (OR = 0.94, 95% CI 0.89,0.99, *P* = 0.04). Also, in the crude model, no association between adherence to the DASH diet and extremely severe stress was seen (OR = 0.96, 95% CI 0.90,1.01, *P* = 0.12), although, after adjustment, a significant association was found (OR = 0.92, 95% CI 0.85,0.99, *P* = 0.02).

## Discussion

We examined the association of the DASH and Mediterranean diet scores on mental health, sleep, and circadian rhythm among women of childbearing age with overweight and obesity in a cross-sectional study. Our results showed that there was a significant inverse association between adherence to the Mediterranean diet and mild and moderate anxiety scores, as well as severe depression. In addition, there was an inverse association between adherence to the DASH diet and the risk of severe depression and extremely severe stress scores. In addition, there was a positive association between adherence to the DASH diet and high odds of completely morning type. Moreover, higher adherence to both dietary scores was associated with good sleep quality.

Previous studies have shown that women are more likely than men to suffer from mental disorders [4]. The reason why we decided to examine the two diets of DASH and Mediterranean in this population with this concern, was that these two dietary scores have several properties that make them healthful diets, particularly their anti-inflammatory and anti-oxidant properties [55, 56]. In the present study, we found an inverse association between following a Mediterranean diet and the odds of severe depression. A study examining the correlation between adherence to the Mediterranean diet and experiencing depressive symptoms among European elderly individuals found that adherence to the Mediterranean diet was inversely associated with depressive symptoms [57]. A meta-analysis indicated that adherence to a diet with high intakes of whole grains, fish, olive oil, vegetables, and fruits and a low intake of animal foods is associated with a risk reduction of depression [52]. Vegetables, fruits, and whole grains are some important foods in the DASH and Mediterranean dietary patterns. Zamani et al. in a study among Iranian women found that adherence to a plant-based dietary pattern was negatively associated with mental disorders [58]. Our study results showed a negative relationship between the Mediterranean diet and the risk of moderate and severe anxiety. A meta-analysis of 13 observational studies expressed that there was no significant association between a vegetarian diet and depression or anxiety [59]; however, different diagnostic criteria were used to assess depression and anxiety symptoms and there was high, unaccounted, heterogeneity among the included studies. Beydoun et al. suggested that oxidative stress may increase the incidence of anxiety and depression [60]. Oxidative stress in the brain can conduct nervous system pathway disturbances, while a meta-analysis found that the DASH diet can help to ameliorate depression [40]. Therefore, the potential role of antioxidants is evident. Moreover, inflammation is another factor that is related to the pathogenesis of anxiety and depression [61]. DASH and Mediterranean diets are rich in whole grains, vegetables, seeds, nuts, and fruit supply which are rich in high amounts of folate and magnesium [61]. Studies have shown that dietary magnesium can reduce inflammation by reducing C-reactive protein, as well as playing a functional role in numerous brain reactions [62]. Also, folate plays an important role in the synthesis of neurotransmitters such as serotonin, norepinephrine, and dopamine [63]. According to a meta-analysis, higher consumption of red and processed meat is associated with higher odds of depression, while red meat and the consumption of its products decrease following a Mediterranean or DASH diet [64].

The previous document showed that individuals with good sleep quality consumed more protein and less fatty foods and high-calorie diets, in comparison to poor sleepers [65]. Moreover, a study among Iranian women showed that adherence to a low-carbohydrate diet was associated with less risk of psychological disorders. This study was performed on patients with type 2 diabetes, who often change their dietary intakes according to nutritionist advice and consider their illness [66]. Epidemiological studies have shown that a higher intake of high-calorie foods and refined carbohydrates is associated with short sleep [67, 68]; moreover, fiber-rich diets can decrease the dietary glycaemic index [69]. Some ingredients and types of foods that are included in the DASH and Mediterranean diet, such as the consumption of higher vegetables, fruits, legumes, and cereals, and lower consumption of fatty and sugary foods, have positive effects on staying asleep and better sleep quality [70]. Another study revealed that greater adherence to the Mediterranean diet was associated with better sleep quality among pregnant women [71]. Also, adherence to plant-based diets was reported to improve body composition, which seems to be beneficial for sleep quality [72]. In line with the mentioned studies, we found that both DASH and the Mediterranean diet are associated with good sleep quality, even after adjustment confounders. Hence, previous studies have shown that sleep restriction is associated with higher BMI and it is well-established that BMI is highly correlated with calorie intake and PA [73, 74]. Although we controlled the effect of BMI on the association of the DASH and Mediterranean diet and the outcomes in the present study, a residual confounding effect was likely to remain.

DASH and the Mediterranean diet are dietary patterns that emphasize the consumption of vegetables and fruits which are high in isoflavones. Studies have shown a positive association between isoflavones intake and sleep quality [75, 76]. For instance, a study among women with type 2 diabetes revealed that dietary total antioxidant capacity was inversely associated with sleep status as well as mental disorders [77]. Another components which are emphasized in the DASH and Mediterranean diet is nuts and plant-based proteins, which are rich in tryptophan as the precursor of serotonin and melatonin. Therefore, a higher intake of nuts and plant-based proteins can support sleep regulation by increasing tryptophan and yielding serotonin and melatonin [78]. Other studies have reported that a higher intake of fats and diets rich in snacks are associated with poor sleep quality [79, 80]. It is plausible that high amounts of animal proteins, including meat can reduce the ratio of tryptophan and tyrosine in circulation, and lead to disturbance of sleep-related neurotransmitters, causing poor sleep quality [81]. Indeed, a significant relationship between circadian rhythm as a completely morning type and relatively evening type, with the DASH diet was reported [82]. In another study, students with an evening chronotype with evening preferences were reported to have lower adherence to the Mediterranean diet, whilst students of the evening type showed a slightly lower consumption of fruits, vegetables, pulses, cereals, and olive oil, and a higher skipping rate for breakfast [83]. Previous studies have shown the importance of breakfast, a meal that is often skipped by people with an evening type [84, 85]. Prior analyses have indicated that the chronotype may be correlated with psychiatric and psychopathological disorders that could affect the dietary pattern, and these disorders are more prevalent in evening-type people [86, 87].

## Strength and limits

The present study has several limitations that should be considered. Because of the cross-sectional nature of the study, we could not extract causal inferences regarding whether poor diet leads to mental and sleep disorders or vice versa. Indeed, psychological disorders could impact eating behavior which is related to anthropometric changes, overweight, and obesity [88]. The DASS-21 is a scale which use to measure the symptoms of depression and anxiety and is not appropriate for clinical diagnosis of depressive disorders. Moreover, FFQ is a self-reported questionnaire that is associated with recall bias and low accuracy. Our findings are not generalizable to the entire population of society, owing to the various health statuses, genders, and other ethnicities and age groups. Moreover, the various phases of the menstrual cycle are related to depressive symptoms [89], which were not considered as a confounder in the present study but should be in subsequent research.

## Conclusion

The current study highlights that the Mediterranean diet score is inversely associated with mild and moderate anxiety, and the DASH diet is inversely associated with extremely severe stress scores and severe depression. Moreover, overweight and obese women who had higher adherence to the DASH and Mediterranean diet scores had better sleep quality. However, further investigations are needed to confirm the veracity of our results. Our findings highlight the need for more research among other populations and for the development of preventative interventions for improving the quality of life of individuals.

## What is already known on this subject?

Previous studies were shown the inverse association between plant-based diets with mental disorders and poor sleep quality. Also, they revealed the inverse association between compliance with the Mediterranean diet and depression and evening chronotype.

## What this study adds?

The results of the present study showed an inverse association between adherence to the Mediterranean and DASH diet with poor sleep quality. Also, the DASH diet was associated with low levels of stress. Mediterranean diet was also associated with lower anxiety levels.
